# Correction: Convective mixing in porous media: a review of Darcy, pore-scale and Hele-Shaw studies

**DOI:** 10.1140/epje/s10189-023-00401-8

**Published:** 2024-01-11

**Authors:** Marco De Paoli

**Affiliations:** 1 Physics of Fluids Group, University of Twente, P.O. Box 217, 7500AE Enschede, The Netherlands; 2 Institute of Fluid Mechanics and Heat Transfer, TU Wien, Getreidemarkt 9, 1060 Vienna, Austria

**Correction to: Eur. Phys. J. E (2023) 46:129** 10.1140/epje/s10189-023-00390-8

Due to technical error, the symbol “\AR” was not shown on pdf, but the symbol has been shown correctly on html version. Now, the symbol has been added back on pdf.

The original article has been corrected.

